# Computational screening of phytochemicals present in some Nigerian medicinal plants against sickle cell disease

**DOI:** 10.1038/s41598-024-75078-w

**Published:** 2024-11-01

**Authors:** Yemisi Elizabeth Asibor, Abel Kolawole Oyebamiji, Dayo Felix Latona, Banjo Semire

**Affiliations:** 1 Department of Pure and Applied Chemistry, Osun State University, Osogbo, Nigeria; 2 Department of Chemistry and Industrial Chemistry, Bowen University, Iwo, Osun State Nigeria; 3 Computational Chemistry Laboratory, Department of Pure and Applied Chemistry, Ladoke Akintola University of Technology, Ogbomoso, Oyo State Nigeria

**Keywords:** Sickle Cell diseases, Bioactive compounds, PASS, ADMET, DFT, Molecular docking, Dynamic simulation, Drug discovery, Anatomy, Diseases, Chemistry

## Abstract

Four hundred Phytochemical (bio-active) compounds having predictive activity for treating Sickle Cell Anemia were screened, using PASS online computational resource. Twenty-six compounds out of the four hundred compounds which showed high probability for treating sickle were further screened for pharmacokinetics profiles (ADMET properties) using SwissAdmet, AdmetSAR 2 and Pro-tox II online resources. Only thirteen compounds that displayed good ADMET properties from the twenty-six were further used for DFT calculations and molecular docking against carbonmonoxy sickle hemoglobin (PDB ID: 5E6E). Molecular docking analysis reinforced by DFT calculations showed that two compounds, phenanthrene-5,6-dione **(A9)** and 2-(3,4-dihydroxyphenyl)-5,7-dihydroxychromen-4-one **(A13, Luteolin)** had the best binding affinity of  − 8.3 and − 8.9 kcal/mol, respectively, compared to voxelotor (GBT-440), a drug use in treating sickle cell disease. Molecular dynamic simulations showed that 2-(3,4-dihydroxyphenyl)-5,7-dihydroxychromen-4-one **(A13, Luteolin)** is highly stable with the protein than voxelotor.

## Introduction

When considering the spectrum of genetic disorders and diseases that impart humanity, only a few can match the multifaceted challenges posed by sickle cell diseases (SCD) and related disorders. Sickle cell disease is a potentially devastating condition resulting from an autosomal recessive inherited hemoglobinopathy. It is a global sickness that transcends national and ethnic boundaries, affecting diverse population worldwide. The molecular basis of sickle cell disease lies in a single base mutation, specifically adenine to thymine, resulting in the substitution of glutamine for valine acid at the sixth codon of the β-globin chain (βS), this leads to high level of 2,3-diphosphoglycerate (2,3-DPG) which interacted with hemoglobin to reduce HbS solubility and promotes polymerization^1^. The creation of a sticky area on the β-globin chain is caused by the substitution of glutamic acid for valine at position 6 on the β-globin molecule^2^. Sickle Cell Disease (SCD) is characterized by a significant burden of illness and a decreased life expectancy primarily attributable to hemolytic anemia and vaso-occlusive processes.

These processes give rise to painful vaso-occlusive episodes, often necessitating hospitalization and contributing to the development of chronic organ damage. Additional complications associated with SCD exhibit considerable variability among individuals, encompassing both acute and chronic issues such as stroke, acute chest syndrome, retinopathy, renal disease, osteonecrosis, cardiovascular problems, and leg ulcers^3,4^. There many recently approved drugs by the FDA employed in the management of sickle cell disease such as voxelotor, crizanlizumab, L-glutamine and hydroxurea. Blood transfusion and bone marrow transplant known as stem cell transplant and gene therapy are also popular remedies^5–7^. In spite of the advances in the realm of public health, these drugs posed many side effects: L-glutamine side effects include nausea, fatigue, chest pain, and musculoskeletal pain. It is only approved by the FDA for patients with 30–65 kg in weight^8^. The side effects of hydroxyurea on the other hand include bone marrow suppression^9^, reduced sperm count, motility, and infertility. In females, it may disrupt the menstrual cycle and affect fertility^10^, kidney damage and impaired renal function^11^, skin rashes, dark patches on skin, vomiting, and dizziness^12^, hair loss, nausea, neutropenia, and oligospermia^13,14^. These drugs are very costly and unaffordable to the sickle cell patients in developing countries especially Sub-Saharan Africa^15^.

Furthermore, problems associated with stem cell transplant and gene therapy are high cost, difficulty in finding suitable donor, and complications wherein the transplanted cells attack the recipient’s organs which usually lead to complications such as seizures, infertility, endocrinopathies, intracerebral hemorrhage, graftversus-host disease (GVHD), and gonadal dysfunction^16–20^. Also, transfusion-acquired infections, iron overload and alloimmunization complications may arise as a result of blood transfusion^21^.

Management of sickle cell disease is a major problem in Sub-Saharan African health sector like Nigeria, where the required haemoglobin S test and medications are not available and too expensive for the most rural dwellers. Since sickle cell cannot be cured but only manage or control, continuous medications which are required are not available and unaffordable; thus poor management of sickle cell disease in Nigeria. To provide alternative treatment and tackle the challenges of unaffordable medications, a close look on the use of phytochemicals from medicinal plants with a possible effect against sickle cell disease, which are abundant in the Sub-Saharan Africa, becomes imperative^13,22^.

Phytochemicals are chemical compounds naturally produced as part of a plant’s metabolic processes. These substances are commonly referred to as “secondary metabolites” and encompass several categories, such as alkaloids, flavonoids, coumarins, glycosides, gums, polysaccharides, phenols, tannins, terpenes, and terpenoids^23,24^. These phytochemicals can be found in a wide array of plants that hold significant importance in both human and animal diets, including fruits, seeds, herbs, and vegetables^25^. Many of these phytochemical constituents are potent bioactive compounds found in various parts of medicinal plants, serving as precursors for the synthesis of valuable drugs^26^. Several medicinal plants native to Nigeria have been reported to have potential efficacy in the treatment of sickle cell disease^27^.

These plants include *Eugenia caryophyllata* (clove, known as “kanunfari” in Hausa), *Piper guineense* (referred to as “eche” in Idoma or “akwa-ose” in Igbo), grains of paradise (*Aframomum melegueta*, locally known as “otuta” in Idoma), *Sorghum bicolor* (notably, the leaf stalk yields an extract resembling blood), *Carica papaya*,* Zanthoxylum zanthoxyloides*,* Moringa oleifera Cajanus cajan* and *Pterocarpus osun*, abundant in Osun state, western region of Nigeria^15,28–31^. These plants are utilized for various health conditions, including the management of sickle cell anemia. They are major herbal components in the traditional Yoruba recipe that forms the basis for the antisickling drug Niprisan^32^.

The anti-sickling potency of *Cajanus cajan* and *Zanthoxylum zanthoxyloides* leaf and seed extracts against sickled erythrocytes hemolysis was carried out, and the results revealed that *Z. zanthoxyloides* leaf, *C. cajan* seed and *C. cajan* leaf extracts decreased the percentage of sickle cells from an initial control level of 91.6–38.2%, 41.7% and 32.8%, respectively^15^.

*Also*,* Z. zanthoxyloides* has been reported to show sickling reversibility, tangible increase in hemoglobin gelling time, and improved rheological properties of drepanocytary blood^33^. In vitro study of extracts from twelve medicinal plants and their antioxidants and anti-sickling activities using spectrophotometric analysis was determined enzymatically and non- enzymatically at different concentrations. All the plants were found capable of inhibiting RBC hemolysis and sickling activity, hemolysis and anti-sickling activity at 250 µg/ml and also possess antioxidants^34^. Methanolic extract of *Petiveria alliacea*, *Canna indica* and *Pergularia daemia* leaves and cationic constituents were evaluated, identified and quantified using emission flame photometry (EFP) and atomic absorption spectrophotometry (AAS). The result showed that leaves extract of *Canna indica* and *Pergularia daemia* at high dose of 300 µg/mL caused significant reductions of sickle red blood cells from 15 to 6% and 15–1%, respectively at 90 min of the antisickling test^35^. In vitro antisickling activities of *Plumbago zeylanica* (Plumbaginaceae) and *Uvaria chamae* (Annonaceae) were evaluated using p-Ihydroxybenzoic acid and normal saline as positive and negative controls. Analysis revealed the presence of flavonoids, saponnins, alkaloids, tannins, cardiac glycosides free and combined anthraquinones; and the extract of *P. zeylanica* had a significantly higher (*p* < 0.05) antisickling activity at the tested concentrations of 10.0, 1.0 and 0.1 mg/ml^36^.

Computational techniques and resources have described as veritable tools that can accelerate discovery and development of new drugs with minima side effects with high potency. Therefore, computational methods would be employed to screen phytochemicals from Nigerian medicinal plants that can lead to rapid identification of new drug candidates which can serve as alternative to orthodox medicine at reduce cost with avoidance of time-consuming laboratory experiments. These aim at tackling limitations connected to the drugs currently in the market by making use of chemical complexity and diversity of phytochemicals which may serve as challenges to traditional drug discovery process.

Density Functional Theory (DFT) is a computational chemistry method that has surged in popularity in recent times for its precise computation of molecular properties. DFT has been widely employed to calculate the electronic structure and attributes of molecules in various states, encompassing both ground and excited electronic configurations, across diverse environments^37,38^. It serves as a formidable instrument for scrutinizing the unchanging aspects of electronic systems, such as their geometrical arrangements and relative energy levels^39,40^. Prediction of Activity Spectra for Substances (PASS), an online web server (http://www.pharm a expe rt.ru/passonline) predicts over 3500 kinds of biological activity, this biological activity includes including pharmacological effects, mechanisms of action, toxic and adverse effects, interaction with metabolic enzymes and transporters influence on gene expression^41^.

Absorption, Distribution, Metabolism, Excretion and Toxicity (ADMET) is an important aspect or stage of drug discovery. It evaluates the safety of the drug candidates. ADMET is very crucial because undesirable pharmacokinetics and toxicity of candidate compounds are the main reason for the failure of drug development. Thus, Prediction of Activity Spectra for Substances (PASS), Absorption, Distribution, Metabolism, Excretion and Toxicity (ADMET), Density Functional Theory (DFT), molecular docking and molecular dynamic simulation would be used in assessing the potency of selected phytochemicals against sickle cell disease. The ADMET profiling, PASS, DFT, molecular docking and molecular dynamic simulations can provide suitable information about the phytochemicals with high potency, better efficacy, less side effects, and strong selectively to specific receptor.

Therefore, the main objective of this work is computationally screening some bio-active compounds from medicinal plants (*Eugenia caryophyllata*,* Piper guineense*,* Aframomum melegueta*, *Sorghum bicolor* and *Pterocarpus osun*) that possesses therapeutic applications in managing sickle cell disease with targeted carbonmoxy sickle hemoglobin in R state conformation (PBD: 5E6E), the polymerization of deoxygenated (T-state) sickle hemoglobin (Hb S) into fibers that distort red blood cells, through PASS, ADMET, DFT, molecular docking and molecular dynamic simulation.

## Materials and methods

### Screening

The canonical smiles of four hundred (400) bioactive compounds were downloaded and save from PubChem database. The canonical smiles of bioactive compounds obtained were subjected to prediction of activity spectra of substances (PASS) web server (www.https:/pharmaexpert.ru.passonline/)^42^ to know if these compounds actually have high probability of treating sickle cell diseases. The compounds that showed probability to be active for treating sickle diseases were subjected to Absorption, Distribution, Metabolism, Excretion and Toxicity (ADMET) and Prediction Toxicity of chemical compounds using Pro-Tox II software. This was evaluated using two ADMET webserves namely SwissADMET (http://lmmd.ecust.edu.cn/admetsar2/result/?tid=684417) and ADMET Sar.2.0 (www.admetexp.org)^43,44^. The Oral bioavailability assessments of the compounds were done using the SwissADME web server (http://www.swissadme.ch/)^45^. The molecular descriptors which describe the anti-sickle activities of the studied compounds evaluated from the optimized structure of the phytochemicals from DFT calculations. DFT with Becke’s three parameter hybrid functional with correlation of Lee, Yang and Parr (B3LYP) was used^46,47^ with 6–31 + G(d, p) basis set was used for the optimization as implemented in Spartan 14 quantum chemistry software^48^.

### Preparation of the ligands

The sickle cell disease inhibitory activities of thirteen selected ligands (A1-A13) that have high probability, less toxicity and good ADMET properties from different natural plants database were studied against the protein in hemoglobin S (carbonmonoxy sickle hemoglobin PDB ID: 5E6E, resolution 1.76Å), while Voxelotor (GBT-440) was used as standard drug for treating SCD. The 3D SDF conformer of all the ligands and standard drug were downloaded from PubChem Database (https://pubchem.ncbi.nlm.nih.gov).

### Preparation of target protein

**Fig. 1:**
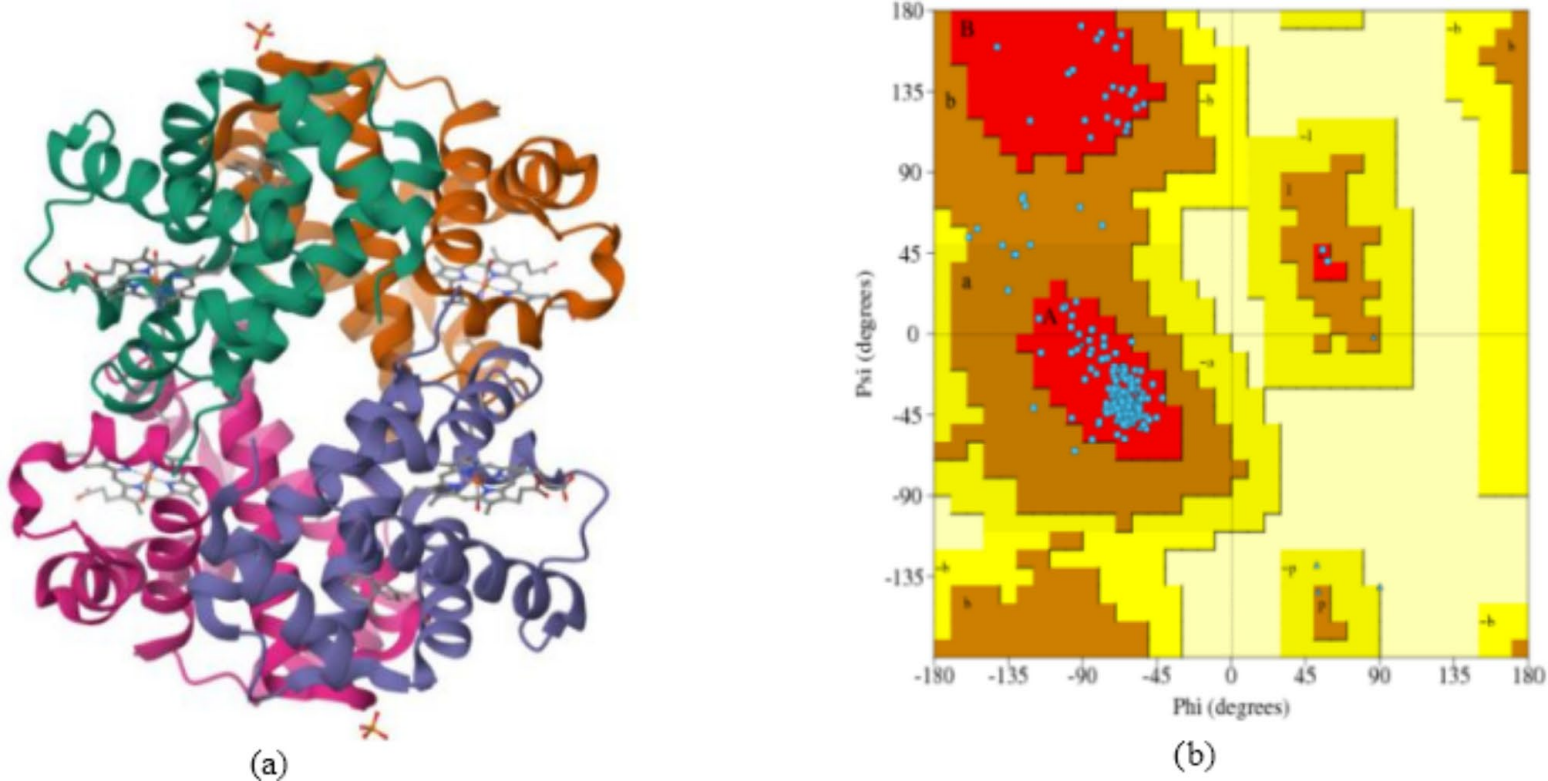
(**a**) crystal structure and binding pocket of carbonmonoxy sickle hemoglobin in R-state conformation (PDB ID: 5E6E) (https://www.rcsb.org/structure/5E6E) and (**b**) Ramachandran plot (PDBsum. www.ebi.ac.uk).

The crystal structures of the protein carbonmonoxy sickle hemoglobin (PDB ID: 5E6E) was downloaded in PDB format from the protein data bank (http://www.rcsb.org/pdb), Fig. 1, with a resolution of retrieved structure as 1.76Å. All water molecules, heteroatoms and unwanted complexes were removed from the crystal structure of the downloaded protein; this was done to ensure that undesired molecular interactions and impurities are avoided, and that no molecules interfered with the potential binding sites of the target proteins during molecular docking. This was done using Discovery Studio Software v.2020. The Ramachandran plot revealed that the receptor was of good quality by using the Volume, Area, Dihedral Angle Reporter (VADAR) webserver. 87% (87%) of the amino acids are in the most favorable region, 11% (11%) are in the unfavorable region and 2% (2%) of the amino acids are disallowed or forbidden.

### Structural and active site analysis of crystal structure of carbonmonoxy sickle hemoglobin in R-state conformation (PDB ID: 5E6E)

The X-ray crystallographic structure of the Crystal Structure of Carbonmonoxy Sickle Hemoglobin in R-State Conformation (PDB ID: 5E6E) as shown in Fig. 1a contains 136 amino acids residue complexed with protoporphyrin IX containing Fe. The Ramachandran plot of the protein reveals that the protein is viable for use (Fig. 1b). The resolution of the protases as revealed by X-ray diffraction was 1.76Å, crystal dimension is a = 53.352 Å, b = 53.352 Å, and c = 191.068 Å, with angles α (90.00), β (90.00) and ϒ (90.00), The R – values (free and work) are 0.243 and 0.193 while the total accessible surface area (TASA) on the protein is 432.6 Å. The final structure contains a dimer (α1β1) in the asymmetric unit, comprising 141 residues in the α-subunit, 146 residues in the β-subunit, 2 heme groups, 2 CO-ligated heme ligands, 2 toluene molecules, a phosphate molecule, and 442 water molecules. Carbonmonoxy Sickle Hemoglobin in R-State Conformation is useful in nitric oxide transport, oxygen transport, blood vessel diameter maintenance, scavenging of heme from plasma and regulation of blood pressure. The active sites in 5E6E are His A 58, Phe A 43, LeuA 29, LeuA 101 and ValA 62^51,52^. This binding pocket, ligand interactions, and all amino acids residues in the active sites of 5E6E, were established using the Computed Atlas for Surface Topography of Proteins (CASTp) (http://sts.bioe.uic.edu/castp/index.html? 2011)^53^ and Biovia Discovery Studio (2019).

### Molecular docking

Molecular docking and binding affinity scores of the optimized ligands and the standard drug against carbonmonoxy sickle hemoglobin (PDB ID: 5E6E) were performed using PxRy and Discovery studio software. The receptor (PDB ID: 5E6E) binding site center and dimension in x, y and z directions generated grid were 35.9837, 31.6925 and 59.0081 for center and 80.5073, 66.1331 and 25.0000 for dimension. The inhibition constant (Ki) µM of the ligands and the standard drug were calculated by employing their binding affinities (∆G) kcal/mol as showed in (Eqs. 1and 2), where R is gas constant, T = 298.15 K (absolute temperature), K_i_ is the inhibition constant and ∆G is the binding energy.

1 $$\Delta G= - nRT\ln {K_{eq}}$$

2 $$K_{i} = \frac{1}{{K_{{eq}} }}$$

### Molecular dynamic simulation

The Molecular Dynamic Simulations was performed for 100 (ns) simulation using GROningen MAchine for Chemical Simulations (GROMACS) tool 2018 version developed by the University of Groningen, Netherlands^54^. Pdb2gmx module was used to generate the required Carbonmonoxy Sickle Hemoglobin in R-State Conformation (PDB: 5E6E) topology file followed by CHARMM36 all-atom force-field selection. phenanthrene-5,6-dione (A9), 2-(3,4-dihydroxyphenyl)-5,7-dihydroxychromen-4-one (A13) topology files were prepared and downloaded from the SwissParam server^55^. A triclinic-shaped water molecules-filled solvation unit cell was built. Addition of Na^+^ and Cl^−^ ions was done for stabilization of the system followed by energy minimization.

Equilibrium setup of the receptor (PDB: 5E6E) complex required and it was done followed by two-step ensembles NVT (constant number of particles, pressure, and temperature) and NPT (constant number of particles, pressure, temperature)^56^. All necessary ensembles deliver control over temperature, pressure coupling provides steadfastness and stabilization of the system during the whole simulation^57^. GORMACS have many inbuilt packages, for the receptor complex Molecular Dynamic Simulations analysis, gmx rms was utilized for Root Mean Square Deviation (RMSD)^58^, gmx rmsf for Root Mean Square fluctuation (RMSF), gmx gyrate for the complete assessment of Radius of Gyration (Rg)^59^ and gmx hydrogen bond for the calculation of numbers of hydrogen-bond (Hbond) formed during the interaction. Lastly, after 100ns molecular dynamic simulations, output trajectory files analyzed and graph plots were generated using Xmgrace program^60^.

## Results

### Prediction of activity spectra of substances database (PASS) for biological activity of the ligands used in treating SCD

The canonical smiles of four hundred (400) bioactive compounds downloaded from PubChem database were subjected to prediction of activity spectra of substances (PASS) web server (www.https:/pharmaexpert.ru.passonline/)^42^ to predict probable anti-sickle cell disease activity of the compounds. Out of the four hundred bio-active compounds, twenty-six compounds (26) showed probability to be active for treating sickle cell diseases as shown in Table 1 along with the standard drug, **Voxelotor (GBT-440)**, and thirteen (13) of these compounds with very good ADMET properties are in parenthesis: Zanthoxytol **(0.161 A1)**, Zingerone **(0.289**,** A2)**, Resveratrol **(0.242**,** A3)**, Scopoletin **(0.229 A4)**, Psoralin, 5-hydroxy **(0.214 A5)**, Isopsoralin **(0.194 A6)**, Pterostilbene **(0.238 A7)**, Phenoxethin **(0.191 A8)**, Phenanthroline-5,6-dione **(0.326 A9)**, Oxyresveratrol **(0.235 A10)**, M-Coumaric acid **(0.236 A11)**, Maesopsin **(0.177 A12)** and Luteolin **(0.182 A13).**

The best three bio-active ligands from the predicted biological activity spectra are in the order: phenanthroline-9,10-dione (0.326) > (4-(4-hydroxy-3-methoxyphenyl) butan-2-one (zingerone, 0.289) > ((E)-3-(3-hydroxyphenyl)prop-2-enoic acid (M-coumaric acid, 0.236). The standard drug, voxelotor (GBT-440) has probability to be active against sickle cell with probability value of 0.155, suggesting that all the thirteen bioactive compounds investigated have higher probabilities than the standard drug. Although, Palmitic acid (0.341), Pentadecanoic acid (0.341), Protocatechuic acid (0.433), Serine (0.308), Phenanthren-9-ol (0.319), Piperazine (0.249), Phenoxathin (0.216), P-Cymene (0.201) and Coumaric acid (0.216) showed higher probability of activity for treating sickle cell than the phenanthroline-9,10-dione, 4-(4-hydroxy-3-methoxyphenyl) butan-2-one and (E)-3-(3-hydroxyphenyl)prop-2-enoic acid compounds, but these compounds failed ADMET rules.

**Table 1:** PASS screening of the bio-active compounds and the standard drug for Sickle Cell.

S/N	Ligands	Probability to be active (Pa)	Probability to be inactive (Pi)
1.	Xylitol	0.183	0.087
2.	Zanthoxylol (A1)	0.161	0.121
3.	Zingerone (A2)	0. 289	0.017
4.	Resveratrol(A3)	0.242	0.036
5.	Scopoletin(A4)	0.229	0.043
6.	Serine	0.308	0.013
7.	Protocatechuic acid”	0.433	0.004
8.	Psoralin, 5-hydroxy-” (A5)	0.214	0.045
9.	Psoralin, n-decanoyl-5-oxo	0.180	0.022
10.	Isopsoralin (A6)	0.194	0.074
11.	Pterostilbene (A7)	0.238	0.038
12.	Piperazine	0.249	0.032
13.	Phenoxazine	0.216	0.053
14.	Phenoxathiin (A8)	0.191	0.077
15.	Phenanthrene	0.320	0.010
16.	Phenanthren-9-ol	0.319	0.010
17.	Phenanthrene-5,6-dione (A9)	0.326	0.009
18.	Pentadecanoic acid	0.341	0.008
19.	Nobilen	0.272	0.022
20.	o-Coumaric acid lactone	0.216	0.053
21.	Palmitic acid”	0.341	0.008
22.	P-cymene	0.201	0.066
23.	Oxyresveratrol (A10)	0.235	0.039
24.	M-coumaric acid“(A11)	0.236	0.039
25.	Maesopsin(A12)	0.177	0.095
26.	Luteolin (A13)	0.182	0.089
27.	Voxelotor (GBT-440) SD	0.155	0.131

### Physiochemical and ADMET properties

Twenty-six compounds with probability of activity for treating sickle cell, as well as voxelotor (GBT-440) drug were subjected to two ADMET profiling using online SwissADMET and ADMET Sar.2.0^43^. SwissADME website was used to compute physicochemical descriptors as well as to predict ADMET parameters such as pharmacokinetic properties, synthetic accessibility, bioavailability score, Veber, Egan, Ghose, druglike nature, medicinal chemistry, water solubility, lipophility^61^ The druglikenss of the twenty-six ligands were estimated using Lipinski’s rules of five, Veber filter, Egan filter, Ghose filter, Muggen filter and bioavailability score. The Lipinski filter or Lipinski’s rule of five (Ro5) is the pioneer rule of five that characterizes small molecules based on physicochemical property profiles which includes hydrogen bond donor (HBD ≤ 5), hydrogen bond acceptor (HBA ≤ 10), molecular weight (150 ≤ MW ≤ 500), lipophilicity (log *P* ≤ 5) and ROTBs ≤ 10^62,63^.

Out of the twenty-six compounds, thirteen ligands obey the Lipinski‘s rule for hydrogen bond donor < 5, hydrogen bond acceptor < 10 and molecular weight from 150 -500 g/mol, TPSA value ≤ 131.6 Å and WLogP value is ≤ 5.88 as displayed in Table 2. All had bioactivity score of 0.55 except M-coumaric acid” with bioactivity score of 0.85. It was evident that the thirteen ligands exhibited high gastrointestinal (GI) tract absorption which makes the ligands suitable as potential drug candidates. The BBB revealed that 2-(3,4-dihydroxyphenyl)-5,7-dihydroxychromen-4-one (A13), 2,4,6-trihydroxy-2-[(4-hydroxyphenyl) methyl]-1-benzofuran-3-one (A12) and 4-[(E)-2-(3,5-dihydroxyphenyl)ethenyl]benzene-1,3-diol (A10) may be able to penetrate blood-brain barrier, this means that the above ligands will have no side effects on the central nervous system (CNS), whereas others including Voxelotor (GBT-440) showed penetration to blood-brain barrier (BBB) which may cause side effects to the central nervous system (CNS)^64^.

The most important task of P-glycoprotein (P-gp) is to keep the central nervous system away from xenobiotics; this leads to increase in the efflux of compounds especially chemotherapeutic agents from the cells^65^. On the other hand, this protein is secreted in some tumor cells and leads to drug-resistant cancers^66,67^. Therefore, it becomes imperative to know if a ligand serves as a substrate to P-gp (i.e., be conveyed out of the cell) or as an inhibitor (to weaken or damage the function of P-gp). All the thirteen studied ligands and standard drug Voxelotor were evaluated to be P-gp non substrate expect these ligands phenoxathiine (A8) and 2,4,6-trihydroxy-2-[(4-hydroxyphenyl) methyl]-1-benzofuran-3-one (A12). In metabolism, A3, A8, A10 and standard drug (Voxelotor) could serve as both non-substrate and inhibitors to CYP3A4, these ligands may cause increase in concentration and overdose of drugs while other compounds have no inhibitory effect on the CYP3A4 enzyme. This means they will have high chance of being converted and hence accessible after oral treatments. All the ligands with Voxelotor inclusive are non-substrate to CYP2D6 and CYP2C9. Ligands A1, A7, A13 and (Voxelotor) are inhibitors to CYP2D6; A3, A7, A10 and (Voxelotor) are inhibitors to CYP2C9 (Table 3); A11 and A12 are non-inhibitors to CYP1A2, and A7 could serve as both inhibitor and substrate to CY2C19 protein. Thus, M-coumaric acid (A11), 2,4,6-trihydroxy-2-[(4-hydroxyphenyl) methyl]-1-benzofuran-3-one (Maesopsin, A12) and Luteolin (A13) have superior metabolic properties than the standard drug^45,68^.

Skin permeability (LogKp) is an important characteristic to be considered when evaluating medicines that may require transdermal delivery^69^. The predicted skin permeation log Kp is − 9.14 cm/s^38,61^. All the ligands had a lesser value compared to the recommended value, which means they all have good skin permeability. Topological Polar Surface Area (TPSA) is the surface area required to bind with the majority of the target receptor and is a good description in characterizing drug absorption bioavailability. The recommended TPSA value is ≤ 131.6 Å^70^. All the ligands had TPSA values less than the recommended (TPSA ≤ 131.6 Å) (Table 2). The synthetic accessibility values of the studied compounds were evaluated based on a scale ranging from 1 (easy to synthesize) and 10 (not easily to synthesis) and these values suggested that the ligands can be easily synthesized as shown in the Table 2. Ligands A9 and A13 showed no pain alert while others has pain alert as shown in the Table 2.

Brain or Intestinal Estimate D permeation method (BOILED-Egg) is an intuitive method to predict simultaneously two key ADME parameters, the passive gastrointestinal absorption (HIA) and brain access BBB^38,61^. Points located in the BOILED-Egg’s yellow represent the analogues predicted to passively permeate the BBB while points in the egg white are relative to the analogues predicted to face passive absorption by the gastrointestinal tract^71^. This implies that compounds A1, A2, A3, A4, A5, A6, A7, A8, A9, A10, A11 can access the brain while A12 and A13 have good gastrointestinal absorption (HIA). The prediction Class (PC) were categorized into Class I and II as fatal if consumed, Class III as toxic if consumed, Class IV as harmful if consumed, Class V perhaps harmful if consumed, Class VI as non- toxic if consumed. All the ligands had their PC III, IV and V (Table 2). Pro-Tox II software predicted that ligands **A1**,** A2**,** A10**,** A11**,** A13** and Voxelotor could be classified as toxicity, with LD_50_ ranging from 2000 ≤ LD_50_ ≤ 5000 mg/kg which may be harmful if swallowed.

**Table 2:** Physiochemical analysis for ligands and the standard drug.

	L	BS	MW	TPSA	LogPo/w	HD	HA	Syn	LD_50_	PC	CY	Mu	I	H	C	Log S	AOT
A1	Yes	0.55	220.31	40.46	3.09	2	2	2.17	3200	5	In	In	In	In	In	-3.3	III
A2	Yes	0.55	194.23	46.30	1.79	3	1	1.52	2580	5	In	In	In	In	In	-1.8	III
A3	Yes	0.55	228.21	60.69	2.48	3	3	2.02	200	3	In	In	In	In	In	-3.6	III
A4	Yes	0.55	192.17	59.67	1.52	4	1	2.62	945	4	In	In	In	In	A	-2.4	III
A5	Yes	0.55	202.16	63.58	1.77	4	1	3.68	500	4	In	A	A	In	A	-2.7	II
A6	Yes	0.55	186.10	43.35	2.21	3	0	3.07	520	4	In	In	A	In	In	-2.9	II
A7	Yes	0.55	250.30	38.69	3.31	3	1	2.29	500	4	In	In	In	In	In	-4.0	III
A8	Yes	0.55	200.26	34.14	3.62	1	0	3.14	1100	4	In	In	In	In	In	-4.5	III
A9	Yes	0.55	208.21	34.14	2.46	2	0	2.33	2000	4	In	A	In	In	In	-3.2	III
A10	Yes	0.55	244.24	80.92	2.08	4	4	2.36	4000	5	In	In	In	In	In	-3.4	III
A11	Yes	0.85	104.16	57.3	1.36	3	2	1.74	2500	5	In	In	In	A	In	-2.2	III
A12	Yes	0.55	288.24	107.22	1.37	6	4	3.09	2000	4	In	In	In	In	In	-3.2	III
A13	Yes	0.55	286.24	111.13	1.73	6	4	3.09	3919	5	In	In	In	In	In	-3.7	III
SD	Yes	0.55	337.37	77.24	2.59	5	1	2.89	4540	5	In	A	In	In	A	-3.7	IV

L = Lipinski, BS = Bioactivity score, GI = Gastrointestinal tract Absorption, LogK = Skin permeamility, MW = molecular weight, SY = Synthetic accessibility, HA = No of hydrogen bond acceptor, HD = No of hydrogen bond donor, LogPo/w = partition coefficient, H = Hepatoxicity, C = Carcinogenicity, I = Immunotoxicity, Mu = Mutagenicity, Cy = Cytotoxocity, AOT = Acute Oral Toxicity, A = Active, In = Inactive, PC = Prediction Class, TPSA = Topological Polar Surface Area.

**Table 3:** ADMET profile of analysis for ligands and the standard drug.

Lig	GI	BBB	CYP3A4 Inh	CYP3A4 Sub	CYP1A2 Inh	CYP1A2 Sub	CYP2C19 Inh	CYP2C19 Sub	CYP2C9 Inh	CYP2C9 Sub	CYP2D6 Inh	CYP2D6 Sub
A1	High	Yes	No	Yes	Yes	No	No	No	No	No	Yes	No
A2	High	Yes	No	No	Yes	Yes	No	Yes	No	No	No	No
A3	High	Yes	Yes	Yes	Yes	No	No	No	Yes	No	No	No
A4	High	Yes	No	No	Yes	Yes	No	No	No	No	No	No
A5	High	Yes	No	No	Yes	Yes	No	No	No	No	No	No
A6	High	Yes	No	No	Yes	Yes	No	No	No	No	No	No
A7	High	Yes	No	Yes	Yes	Yes	Yes	Yes	Yes	No	Yes	No
A8	High	Yes	Yes	Yes	Yes	Yes	Yes	No	No	No	No	No
A9	High	Yes	No	Yes	Yes	No	Yes	No	No	No	No	No
A10	High	No	Yes	Yes	Yes	No	No	Noz	Yes	No	No	No
A11	High	Yes	No	No	No	No	No	No	No	No	No	No
A12	High	No	No	No	No	No	No	No	No	No	No	No
A13	High	No	Yes	No	Yes	No	No	No	No	No	Yes	No
SD	High	Yes	Yes	Yes	Yes	No	Yes	No	Yes	No	Yes	No

Inh = Inhibitor and Sub = Substrate.

### Molecular docking analysis

The docking simulations of the DFT optimized structures of the compounds were carried out against Crystal Structure of Carbonmonoxy Sickle Hemoglobin in R-State Conformation (PDB ID: 5E6E) and the conformation in each ligand-receptor complex with highest free energy of interactions was considered as best and most suitable conformation. A potential active drug is expected to have inhibitory values from 0.1 and 1.0µM and it should not be greater than 10nM^72^. The binding affinity/scoring energy ranged from − 6.1 to -9.1 kcal/mol and the Inhibition constant ranged from 0.25 to 33.60 (µM). The molecular docking results were showed on Tables 4 and 5.

**Fig. 2:**
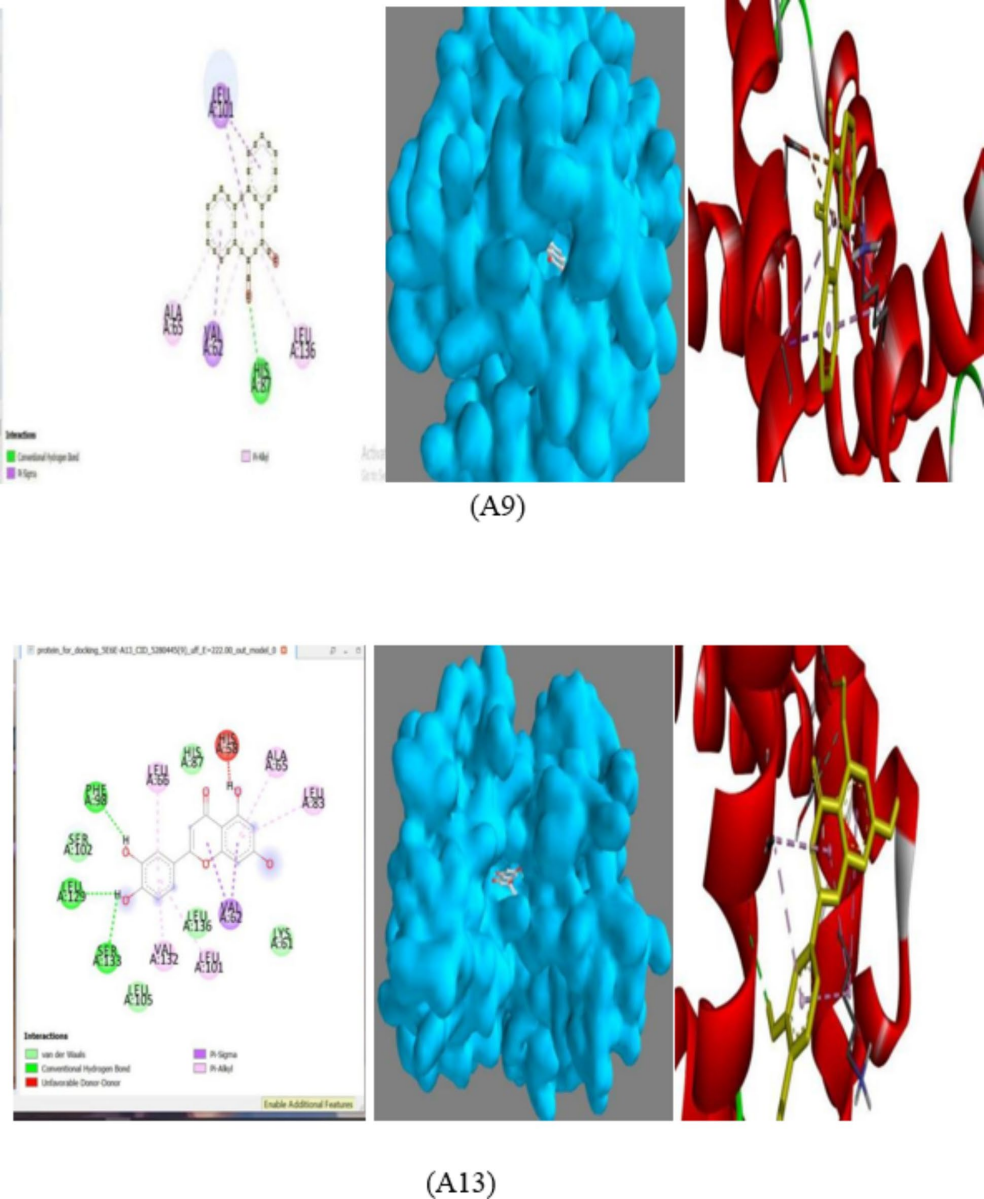
2D binding interactions, binding surface and, 3D binding interactions A9 and A13. (Biovia Discovery Studio Visualizer 2021, PyRx-Python Prescription 0.8).

Phenanthrene-5,6-dione (A9) had − 9.1 kcal/mol, 2-(3,4-dihydroxyphenyl)-5,7-dihydroxychromen-4-one (A13) had − 8.9 kcal/mol with the target protein (PDB ID: 5E6E) while the standard drug Voxelor had − 7.4 kcal/mol. Phenanthrene-5,6-dione (A9) had Pi-Sigma interaction with the protein (PDB ID: 5E6E) via LEU 101 and VAL 62. Pi- Alkyl interaction with the protein (PDB ID: 5E6E) via LEU 136 and ALA 65. Conventional hydrogen bond interaction with the protein (PDB ID: 5E6E) via HIS 86 as shown in the Fig. 2. 2-(3,4-dihydroxyphenyl)-5,7-dihydroxychromen-4-one (A13) had Pi-Sigma interaction with the protein (PDB ID: 5E6E) via VAL 62, Pi- Alkyl with LEU 66, VAL 132, ALA 65, and LEU 83, Pi-Sigma interaction with VAL 62, Van Der Waaals interaction with SER 102, HIS 87, LEU 136, LYS 61, LEU 105, conventional hydrogen bond interaction with PHE 98, LEU 129, SER 133 and unfavorable donor-donor interaction with HIS 58 (Fig. 2).

**Table 4:** Showing the binding affinity and inhibition constants of 5E6E receptor with the ligands.

Ligand	Binding affinity ΔG (kcal/mol)	Inhibition constants Ki (µM)
A1	-6.6	14.45
A2	-6.9	8.70
A3	-6.1	33.60
A4	-6.1	33.60
A5	-6.4	20.25
A6	-7.9	1.60
A7	-6.2	28.38
A8	-7.2	5.24
A9	-9.1	0.25
A10	-7.6	2.67
A11	-6.6	14.45
A12	-7.5	3.16
A13	-8.9	0.29
Voxelor	-7.4	3.74
Coligand (Protoporphyrin IX containing Fe)	-8.6	0.49

**Table 5:** Binding affinity and non-bonding interactions of 5E6E receptor with the ligands.

IUPAC NAME	Binding affinity ΔG (kcal/mol)	Inhibition constant Ki (µM)	5E6E receptor amino acids forming H bond with ligands	H-bond distance (Å)	Electrostatic/Hydrophobic interactions involved
furo[2,3-h]chromen-2-one (A6)	-7.9	1.60	ALA 130, SER 102		LYS 99, HIS 103, LEU 106, ASP 126,
Phenoxathiine (A8)	-7.2	5.24			ALA 130 HIS 103, ASP 126, SER 102 LYS 99,
phenanthrene-5,6-dione (A9)	-9.1	0.25	HIS 87		LEU 101, LEU 136, ALA 65, Val 62, HIS 87
4-[(E)-2-(3,5-dihydroxyphenyl)ethenyl]benzene-1,3-diol (A10)	-7.6	2.67	LEU 129		PHE 43, TYR 42, VAL 93, LEU 101,
2-(3,4-dihydroxyphenyl)-5,7-dihydroxychromen-4-one (A13)	-8.9	0.29	PHE 98, LEU 129, SER 133	2.45, 1.93	HIS 58, LEU 66, PHE 98, LEU 129, SER 133, ALA 65, LEU 83, VAL 132, VAL 62, LEU136, LEU 101 HIS 87

### Geometries of calculated molecular description of the studied ligands

The thirteen phytochemicals (A1-A13) with good ADMET properties and Voxelotor (GBT-440) were optimized using Density Functional Theory (DFT) at ground state in water to calculate the reactivity descriptor of the compounds. The Density Functional Theory (DFT) of Beckes’s three-parameter hybrid functional using the Lee, Yang and Parr correlation functional (B3LYP) with 6–31G (d, p) basis set was used for the geometry optimization and energy calculation. The calculated molecular descriptions are the highest occupied molecular orbital (HOMO), the lowest unoccupied molecular orbital (LUMO), dipole moment (DM), Band Gap $({E}_{g}=\:{E}_{HOMO}-{E}_{LUMO})$, electron affinity $(EA = -{E}_{LUMO})$, ionization potential $(IP = -\:{E}_{HOMO})$, chemical potential $(\mu = \:\frac{IP+EA}{2})$, chemical hardness $(\eta = \:\frac{IP-EA}{2})$, chemical softness $(\text{S}=1/2{\upeta\:})$, electron donating power $(\:{\omega\:}^{-}=\frac{{\left(3IP+EA\right)}^{2}}{16\left(IP-EA\right)})$, electron accepting power $(\:{\omega\:}^{+}=\frac{{\left(IP+3EA)\right)}^{2}}{16\left(IP-EA\right)})$, global electrophilicity index $(\omega = \:\frac{{\left(IP+EA\right)}^{2}}{4\left(IP-EA\right)})$) and ∆$\:{\omega\:}^{\mp\:}\:({\omega\:}^{+}-{\omega\:}^{-})$ as presented in Table 6. The frontier orbital energies (the HOMO and LUMO) govern chemical reactivity of a molecule in which the HOMO and LUMO denoting electron donating and accepting capability, respectively, of a molecule. Thus, the high value of HOMO energies enhances the ligand’s capacity bind with receptor^73^.

The band gap ($\:{E}_{g}$) is energy difference between the highest energy level containing electrons (HOMO) and the lowest energy level that is unoccupied (LUMO) in a molecule’s electronic structure. A high ($\:{E}_{g}$) indicates higher kinetic stability and low reactivity, while a smaller ($\:{E}_{g}$) facilitates the transfer of electrons within a molecule, leading to potential changes in its charge distribution and properties^74^. A9 had the lowest ($\:{E}_{g}$) value, Voxelotor had the highest value which indicated that A9 is the most reactive ligand while Voxelotor is the least reactive (Table 6).

**Fig. 3:**
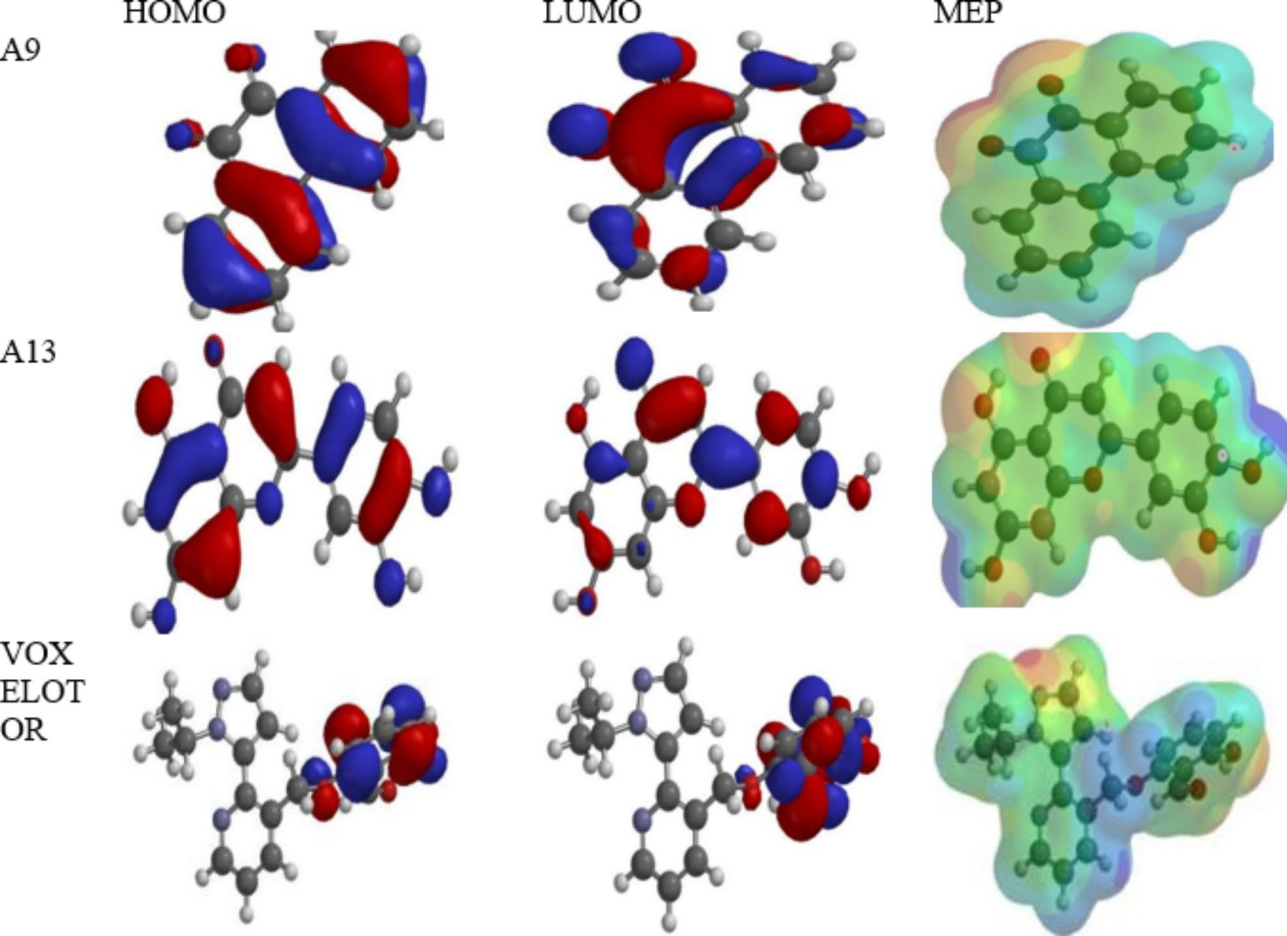
The frontier orbitals and molecular electrostatic potential (MEP) diagrams for A9, A13 and Voxelotor (GBT-440) (Wavefunction/Spartan 14 version 1.1.4, www.wavefun.com).

The HOMO and IP energies show that **A13** can easily release electrons to the surrounding receptor and also exhibits strong interactions than the **A9** and standard drug (Voxelotor (GBT-440). Also, µ, ω, ∆$\:{\omega\:}^{\mp\:}$ and dipole moment values as shown in Table 6 support stronger stability of **A13** in the protein-ligand complex, stronger electron accepting capability and stronger non-bonding interactions between the ligand and protein. The MEP diagram shows that 2-(3,4-dihydroxyphenyl)-5,7-dihydroxychromen-4-one (**A13)** about four nucleophilic centers on hydroxyl oxygen atoms which may enhance its interactions with the receptor (Fig. 3). The HOMO, IP, ω and ∆ $\:{\omega\:}^{\mp\:}$ values suggested that **A9** and the standard drug may have similar energetic interactions with the protein, which is agreement with the calculated docking affinity.

**Table 6:** Optimization of the ligands and drugs in water.

COMP	HOMO eV	LUMO eV	DM Debye	Eg	µ	η	IP	EA	ω	∆$\:{\omega\:}^{\mp\:}$
A1	-5.68	-0.04	2.35	5.64	-2.86	2.82	5.68	0.04	1.44	-3.60
A2	-5.50	-0.28	3.63	5.22	-2.89	2.61	5.50	0.28	1.59	-3.85
A3	-5.31	-1.28	2.88	4.03	-3.29	2.02	5.31	1.28	2.68	-5.89
A4	-5.68	-1.58	9.78	4.10	-3.63	2.05	5.68	1.58	3.21	-6.95
A5	-5.79	-1.56	7.04	4.23	-3.67	2.12	5.79	1.56	3.17	-6.91
A6	-6.12	-1.49	5.27	4.63	-3.80	2.32	6.12	1.49	3.11	-6.83
A7	-5.42	-1.07	0.18	4.35	-3.25	2.18	5.42	1.07	2.42	-5.39
A8	-5.57	-0.49	1.43	5.08	-3.03	2.54	5.57	0.49	1.80	-4.25
A9	-6.26	-2.69	5.98	3.57	-2.48	1.79	6.26	2.69	1.72	-4.37
A10	-5.38	-1.28	1.69	4.10	-3.33	2.05	5.38	1.28	2.70	-5.92
A11	-6.11	-1.65	4.03	4.46	-3.88	2.23	6.11	1.65	3.37	-7.30
A12	-5.84	-1.58	3.07	4.26	-3.71	2.13	5.84	1.58	3.08	6.99
A13	-5.74	-1.65	6.28	4.09	-3.69	2.05	5.74	1.65	3.30	-7.19
Voxelotor	-6.48	-0.08	3.60	6.40	-3.28	3.2	6.48	0.08	1.60	-4.16

HOMO = Highest occupied molecular orbital, LUMO = Lowest unoccupied molecular orbital, DM = Dipole moment, Eg = Band Gap, µ = Chemical potential, η = Chemical hardness, IP = Ionization potential, EA = Electron affinity, ω = Global electrophilicity index, ∆$\:{\omega\:}^{\mp\:}$ = Net electrophilicity index.

### Molecular dynamic simulation (MDS)

**Fig. 4:**
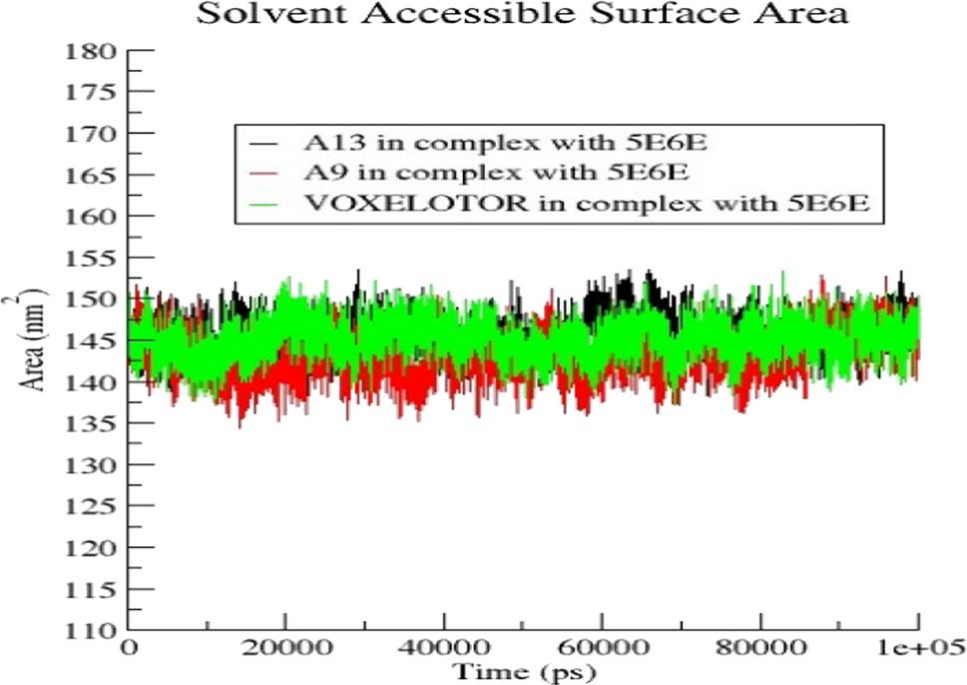
Solvent accessible surface area plot of phenanthrene-5,6-dione (A9), 2-(3,4-dihydroxyphenyl)-5,7-dihydroxychromen-4-one (A13), and Voxelotor with 5E6E.

**Fig. 5:**
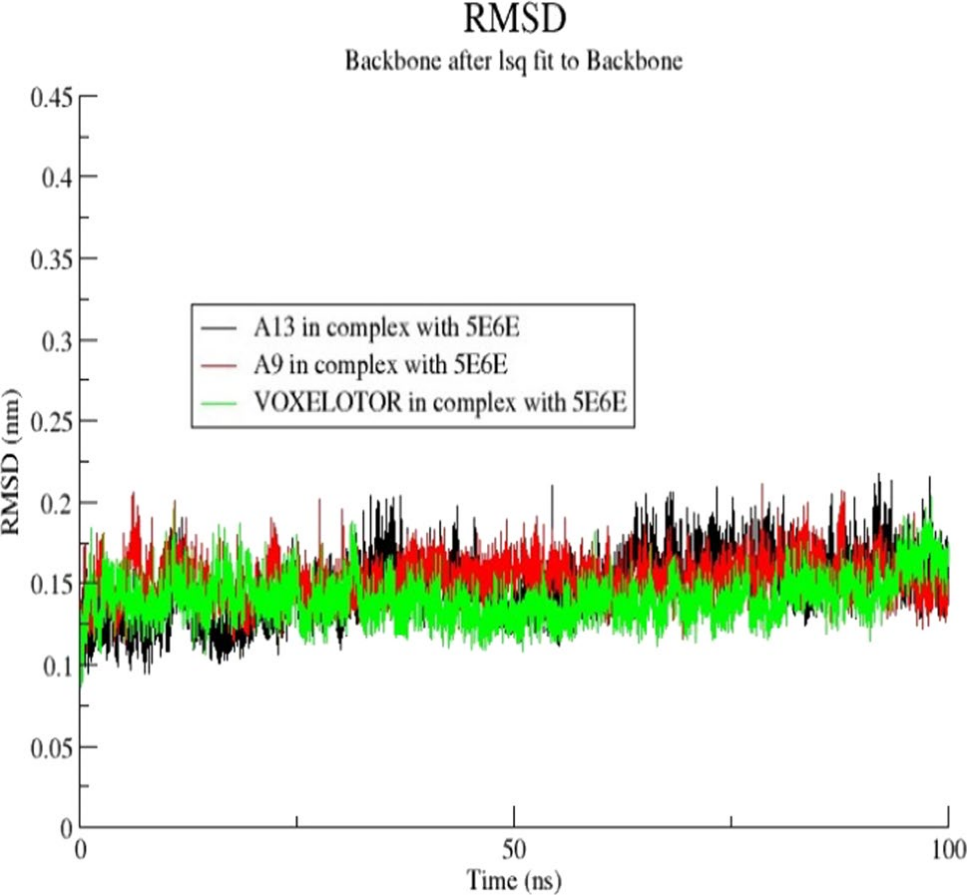
Graphical representation showing RMSD plot of phenanthrene-5,6-dione (A9), 2-(3,4-dihydroxyphenyl)-5,7-dihydroxychromen-4-one (A13), and Voxelotor with 5E6E.

**Fig. 6:**
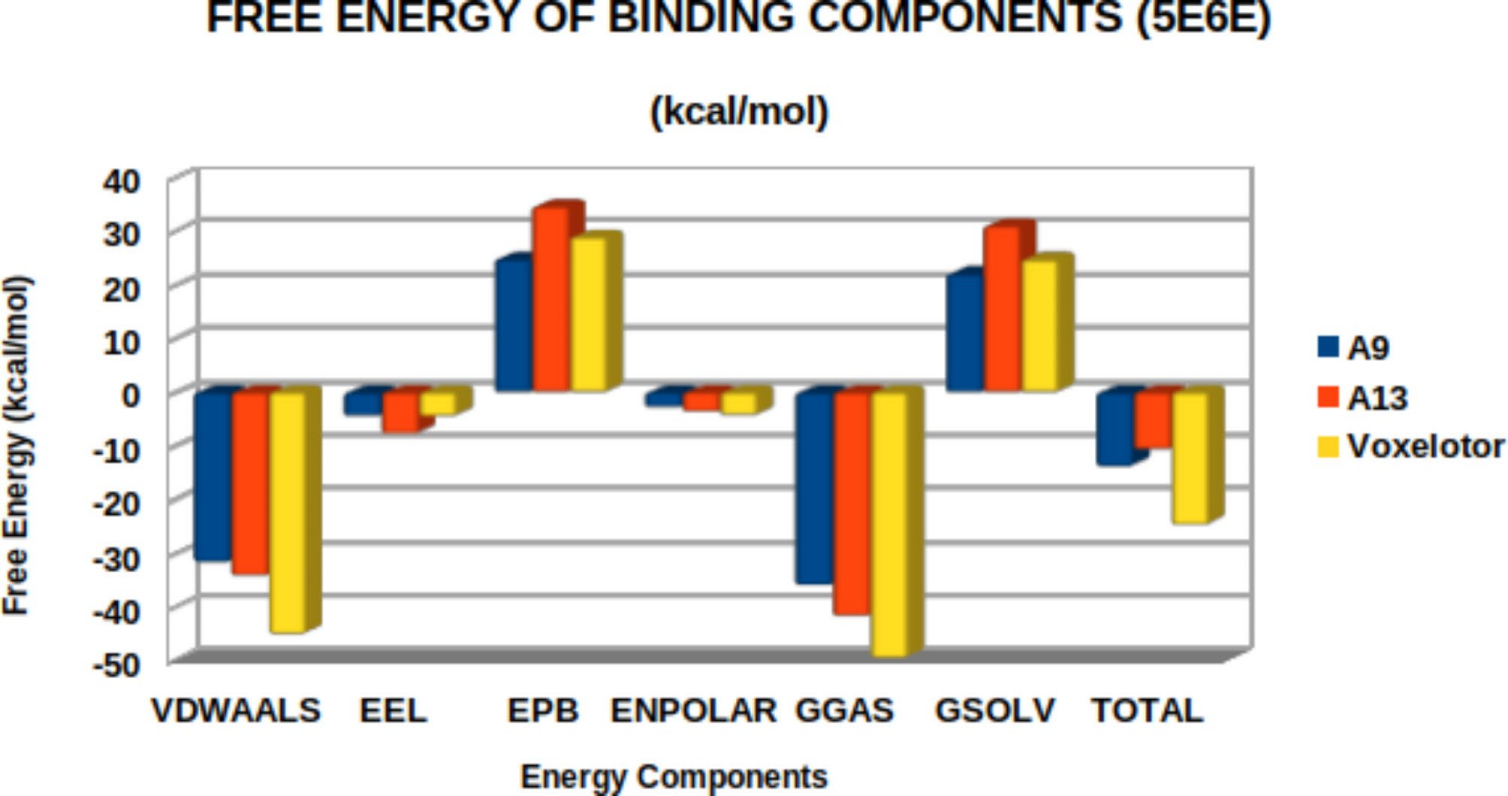
Free energy of binding components plot of phenanthrene-5,6-dione (A9), 2-(3,4-dihydroxyphenyl)-5,7-dihydroxychromen-4-one (A13), and Voxelotor with 5E6E.

The molecular docking analysis supported by DFT results was further investigated by performing molecular dynamic simulation study. This was done to predict the actual binding stabilities between ligands phenanthrene-5,6-dione (A9), 2-(3,4-dihydroxyphenyl)-5,7-dihydroxychromen-4-one (A13) and the standard drug) and Carbonmonoxy Sickle Hemoglobin in R-State Conformation (PDB ID: 5E6E). Molecular dynamic simulation study was done to understand the positions of different atoms and molecules during the binding of the ligands with the receptors^75^. Furthermore, 100ns MDS experimentation observed parameters like Solvent Accessible surface area, RMSD, and free energy binding component plot interpretation, revealed deviation and fluctuation of **5E6E** during the simulation. The root mean square deviation (RMSD) provides a measure of the average distance between the atoms of the protein–ligand complexes. The RMSD values were in the range of 0.1–2.3 nm throughout the period for all complexes. The 5E6E complexation with 2-(3,4-dihydroxyphenyl)-5,7-dihydroxychromen-4-one **(A13)**, showed a stable pattern as compared to that of phenanthrene-5,6-dione **(A9)** (Fig. 4). The observed RMSD values for phenanthrene-5,6-dione **(A9)**, 2-(3,4-dihydroxyphenyl)-5,7-dihydroxychromen-4-one (A13) and standard drug Voxelotor (GBT-440) complexed with **5E6E** were between 0.12 and 0.17 nm and gained more stability from 0 to 100 ns as showed on Fig. 5. Van der Waals calculations, electrostatic potential energy and polar solvation, of the ligands phenanthrene-5,6-dione **(A9)**, 2-(3,4-dihydroxyphenyl)-5,7-dihydroxychromen-4-one2-(3,4-dihydroxyphenyl)-5,7-dihydroxychromen-4-one **(A13)** and voxelotor with the target (PDB: 5E6E) were shown in the Fig. 6. The binding free energy of the ligand (TOTAL) was calculated by subtracting the free energies of the receptor and ligand from the complex. For each of the complex, the total free energy was the sum of the phase energy (GGAS) and the solvation energy (GSOLV). GGAS was contributed mainly from van der Waals interactions (VDWAALS) and electrostatic interactions (EEL), and GSOLV was the sum of polar (EPB) and non-polar (ENPOLAR) interactions (Table 7).

**Table 7:** Decomposition of free energy components for 5E6E.

Lig	VDWAALS	EEL	EPB	ENPOLAR	GGAS	GSOLV	TOTAL
A9	-31.61 ± 0.03	-4.36 ± 0.06	24.88 ± 0.05	-2.69 ± 0.00	-35.96 ± 0.06	22.18 ± 0.05	-13.78 ± 0.05
A13	-34.09 ± 0.04	-7.62 ± 0.08	34.73 ± 0.08	-3.58 ± 0.00	-41.71 ± 0.09	31.15 ± 0.08	-10.56 ± 0.07
Voxelotor	-45.01 ± 0.05	-4.42 ± 0.06	29 ± 0.06	-4.2 ± 0.00	-49.43 ± 0.08	24.8 ± 0.06	-24.63 ± 0.07

VDWAALS = van der Waals interactions, EEL = Electrostatic interaction, EPB = Polar interactions, ENPOLAR = Non - polar interactions, GGAS = Gas phase energy, GSOLV = Solvation energy, TOTAL is the binding free energy of the ligand.

## Discussions

ADMET (Absorption, Distribution, Metabolism, Excretion, and Toxicity) screening tools provide predictions about the pharmacokinetic and toxicity profiles of the phytochemicals. The findings from the phytochemicals screening can help in identification and optimization of lead compounds with the most favorable pharmacokinetic and pharmacodynamic properties for preclinical studies, which include assessment of the lead compounds therapeutic potential though in vitro and in vivo studies. Clinical trials are essential for safety, efficacy, and tolerability of the lead compounds in humans. Clinical trials are conducted in several phases, starting with Phase I (safety and dosage), followed by Phase II (efficacy and side effects), and Phase III^49^. The challenges that can be encountered in translating the findings to clinical applications are complexity of phytochemicals, variability in biological activity, and intellectual property and commercialization. Future research direction can include experimental validation and optimization of lead compounds, advanced formulation technique, clinical feasibility studies, and multi-target and synergistic effects.

The limitations of computational methods include extent of mathematical approximations, algorithms and scoring functions used which may lead to inadequate sampling of conformational space and assumptions about molecular interactions, inability to fully capture dynamic nature of proteins or the impact of water molecules, ions, and other factors present in vivo. Therefore, integration of the experiment with computational simulation is most often needed to assist in enriching the interpretation, providing new detailed molecular understanding of the systems. Despite these shortcomings, the use of computer modeling and simulations is becoming more and more necessary to understand biological mechanisms and to create drugs with desired binding and dissociation kinetics properties that have recently been discovered as critical to pharmacological effectiveness and safety^49,50^.

## Conclusion

A conventional or traditional clinical trial method takes a significant expenditure in time, money and resources, and it is also possible that such drug candidate fails. Molecules which involves in the clinical trials such as chemical entity, orphan molecule and drug candidates need to undergo in-silico modelling in order to confirm the physiochemical properties, drug-ability and their atomicity level. In this study, twenty six (26) potential compounds for managing sickle cell disease were evaluated for their drug-likeness in treating sickle cell disease using PASS, ADMET, DFT Molecular docking and Molecular dynamic simulation. Phenanthrene-5,6-dione **(A9)** was reported to be found in *Moringa lucida*, a plant that has efficacy in treating sickle cell diseases. 2-(3,4-dihydroxyphenyl)-5,7-dihydroxychromen-4-one **(A13**,** Luteolin)** is a phytochemical found in *Cajanus Cajan* seeds, this plant was reported in the literature to possess anti-sickling properties. *Cajanus Cajan* was reported to assist in reduction of polymerization rate, reverse sickle red blood cell, increase the oxygen affinity of Hb. The results obtained from the in-silico analysis showed that compounds phenanthrene-5,6-dione **(A9)** and 2-(3,4-dihydroxyphenyl)-5,7-dihydroxychromen-4-one **(A13**,** Luteolin)** has higher potential in treating sickle cell disease than the standard drug voxelotor (GBT-440). 2-(3,4-dihydroxyphenyl)-5,7-dihydroxychromen-4-one is a flavonoid and a rutin phytochemical and phenanthrene-5,6-dione (A9) is an anthraquinone. Further research can be done on the derivatives of these compounds.

## Data Availability

All data generated or analyzed during this study are included in this article.
